# Seasonal Nutritional Profile of *Gelidium corneum* (Rhodophyta, Gelidiaceae) from the Center of Portugal

**DOI:** 10.3390/foods10102394

**Published:** 2021-10-09

**Authors:** Mário Cavaco, Adriana Duarte, Marta V. Freitas, Clélia Afonso, Susana Bernardino, Leonel Pereira, Mendelson Martins, Teresa Mouga

**Affiliations:** 1 MARE—Marine and Environmental Sciences Centre, ESTM, Polytechnic of Leiria, Edifício CETEMARES, Av. Porto de Pesca, 2520-641 Peniche, Portugal; mariopalosc@hotmail.com (M.C.); adrianajesusduarte.96@gmail.com (A.D.); marta.freitas@ipleiria.pt (M.V.F.); susana.bernardino@ipleiria.pt (S.B.); mougat@ipleiria.pt (T.M.); 2 MARE—Marine and Environmental Sciences Centre, Department of Life Sciences, University of Coimbra, Calçada Martim de Freitas, 3000-456 Coimbra, Portugal; leonel.pereira@uc.pt; 3 ESTM—School of Tourism and Maritime Technology, Polytechnic of Leiria, Rua do Conhecimento 4, 2520-614 Peniche, Portugal; mendelmartins@outlook.pt

**Keywords:** red seaweeds, total protein, carbohydrates, total lipids, heavy metals, antioxidant capacity, seasonal variations

## Abstract

*Gelidium corneum* is a well-known agarophyte, harvested worldwide for its high agar quality. However, the species also exhibits an interesting nutritional profile, but with seasonal variations. Therefore, to evaluate the nutritional value of *G. corneum*, ash, crude protein, total lipids, and carbohydrates were analyzed at different times of the year. The heavy metals mercury, arsenic, lead, cadmium, and tin, as well as iodine were also measured. Finally, the seasonal antioxidant capacity of *G. corneum* extracts was evaluated. Our results indicate that the biomass is rich in protein (up to 16.25 ± 0.33%) and carbohydrates (up to 39.5 ± 3.29%), and low in lipids (up to 2.75 ± 0.28%), and especially in the summer, the AI, TI indexes, n-6/n-3 and h/H ratios (0.93, 0.6, 0.88 and 1.08, respectively) are very interesting. None of the contaminants exceeded the legally established limits, and the iodine values were adequate for a healthy diet. Finally, the antioxidant capacity is fair, with the DPPH ≤ 10.89 ± 1.46%, and ABTS ≤ 13.90 ± 1.54% inhibition, FRAP ≤ 0.91 ± 0.22 AAE.g^−1^, and TPC ≤ 6.82 ± 0.26 GAE.g^−1^. The results show that *G. corneum* is an attractive resource, with potential use as food or as a food supplement.

## 1. Introduction

The marine environment is home to some of the richest and most complex ecosystems, representing a wide genetic range within the species found in this biota [1]. Whilst plants are known to be the primary producers on land, marine algae have the same function in the ocean, estimated in 2012 by Guiry [2] to a total of 350,000 species. They can be found in coastal areas, providing habitat or food to other marine organisms, and in tropical, temperate, or artic zones from the intertidal zone to depths reaching 250 m [3,4,5,6]. Among these species, seaweeds represent those that are macroscopic, with visible, developed thalli. Seaweeds are classified according to their predominant accessory pigment. Chlorophyll *b*, fucoxanthin, and phycoerythrin are the predominant pigments for the green (Chlorophyta), brown (Phaeophyceae), and red seaweeds (Rhodophyta) respectively [7,8].

These organisms produce the necessary energy for their metabolism through photosynthesis and store the resulting chemical energy as organic compounds. Many of these exhibit interesting properties, such as antioxidant, antibacterial, and anti-tumoral activities, to name a few [9,10]. As a result, algae are used due to their biotechnological potential and possible applications, namely, in the food, cosmetics, and pharmaceutical industries [11].

Seaweeds have been part of the human diet since at least 600 BC and have been consumed as a vegetable since prehistoric times in East Asia. This ancient tradition has raised interest in the scientific community towards algae, resulting in many studies that establish the health benefits linked to their consumption [12,13,14,15,16,17,18].

Like most seaweeds, red algae are made up of large amounts of polysaccharides, many of which exhibit the behavior of non-soluble fibers. This means that they are long chain molecules that are not digestible by the human body, and therefore have no caloric value. Nonetheless, they are high in total fiber (10–75%), which can reduce the risk of colon cancer and obesity [19,20]. Red seaweeds also show a relatively high protein content, reaching values that can reach 47% in *Porphyra*/*Pyropia*. Although in other species the protein content may not be as abundant, they are of higher quality than proteins from plant sources such as rice, wheat, and beans, notably in the presence of essential amino acids [21,22]. Moreover, the supplementation of algae in food is associated with the growth and maintenance of beneficial intestinal flora, a reduction in the risk of colorectal cancer, and a significant reduction in breast cancer due to high bioavailability of dietary iodine [23].

Regarding lipid content, seaweeds are generally low in lipids, but within these algae, they are rich in polyunsaturated fatty acids (PUFA) that are essential for human and animal nutrition, specifically Omega-3 (n-3) and Omega-6 (n-6) fatty acids [24]. These fatty acids are considered essential, as the human body cannot synthesize them naturally, requiring supplements in the diet. Omega-3 fatty acids have anti-inflammatory activity, which is related to reducing the severity of asthma, influencing the blood clotting process, and proper developing and functioning of the brain and retina [25,26,27]. Lauric (12:0), myristic (14:0), myristoleic (14:1), palmitic (16:0), palmitoleic (16:1), stearic (18:0), oleic (18:1), and linoleic (18:2) acids are the most common fatty acids present in red macroalgae. These are also known to have high concentrations of arachidonic (20:4 n-6) and eicosapentaenoic (20:5 n-3) acids, which play a role in bioregulation of cellular processes [28,29]. 

Seaweeds are considered edible due to their healthy nutritional profile and the lack of endogenous toxins [30]. Yet, they tend to accumulate large amounts of heavy metals, namely lead, cadmium, and arsenic, and many other minerals, from the environment. Hence, the quality of the water in which seaweed grows is a relevant factor for the quality of biomass when it is being regarded as food [31]. 

**Figure 1:**
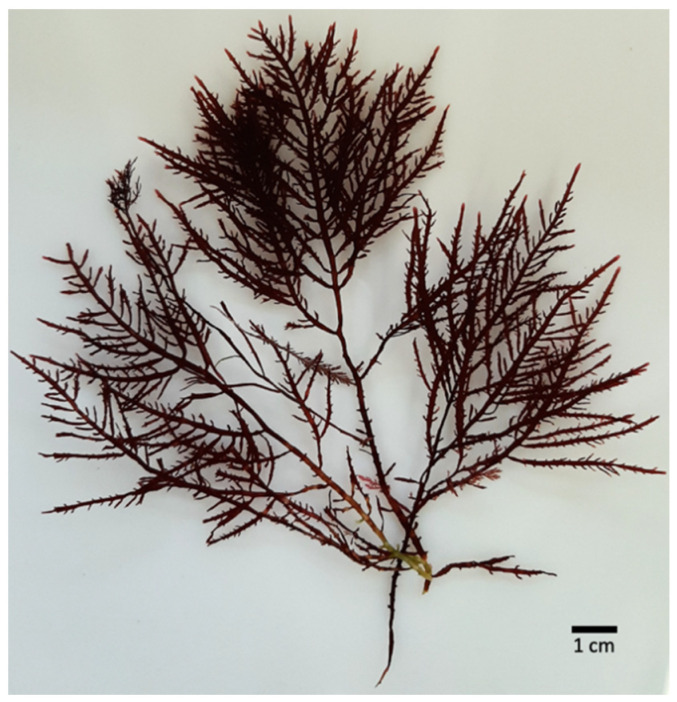
Macroscopic image of *Gelidium corneum*.

Five major genera of red seaweeds are considered edible: *Palmaria*, *Gracilaria*, *Gelidium*, *Porphyra*/*Pyropia*, and *Kappaphycus*/*Eucheuma* [32,33]. *Gelidium corneum* (Hudson) J.V. Lamouroux, 1813, known as Atlantic Agar, is widely distributed from the Indian to the Atlantic Oceans [34]. Formerly known as *G. sesquipedale*, it is a perennial Rhodophyta, inhabiting the coastal area at shallow depths, always attached to a solid substrate, in cold waters that have high sunlight exposure. Its fronds are large, red, and shiny with a cartilaginous texture, cylindrical at the base and flattened at the top, branching in the upper half, through secondary and tertiary branches, usually opposite (Figure 1) [35,36,37]. It is traditionally used for agar extraction as it produces the highest quality gelling agent [38].

Although the genus *Gelidium* is not yet recognized as food by the European Union [39] it is accepted for human use by the U.S. Food and Drug Administration [40,41]. It is also worth noting that traditionally several species of *Gelidium* have been used as food in different parts of the world, including *G. corneum* [17].

Abiotic factors such as fluctuating nutrient availability, UV radiation, temperature, and salinity variations, among others, can trigger environmental stress that triggers metabolic changes, which in turn can lead to fluctuations in the production of primary and secondary metabolites [42]. Reactive oxygen species (ROS) may be produced during these biochemical processes. These ROS are highly reactive with other molecules due to their one or more unpaired electrons and, if these compounds accumulate, they can lead to an imbalance in the body’s ability to tackle the harmful oxidant effects of the free radicals [43]. This is known as oxidative stress, and its influence depends on the balance of ROS production and antioxidant defenses. The mechanism underlying the protection from oxidative stress in an organism by antioxidant compounds is based on the removal and scavenging of free radicals and their precursors [44]. Seaweeds are among the most interesting organisms in the production of antioxidant compounds, which prevent oxidative stress. These compounds have a beneficial effect on human health when seaweeds are consumed as food [45,46].

So far, synthetic antioxidants are commercially available, but their use and applications are limited due to possible side effects, such as gastrointestinal problems [47]. 

Due to rising interest in natural and seafood products, the present study aimed to gather data regarding the nutritional profile of *G. corneum* to provide clear information on its potential as a food ingredient, while considering the seasonal variations of the primary compounds. Accordingly, the contents of ash, carbohydrates, lipids, and proteins, as well as the lipid profile were evaluated. Being an important food additive, the seasonal agar content was also determined. Furthermore, in order to assess the level of toxic contaminants in the collected biomass, levels of heavy metals (arsenic, cadmium, lead, and mercury) as well as iodine were determined. Finally, the antioxidant capacity of the biomass, collected in different seasons of the year, was evaluated to determine the potential use of *G. corneum* as functional food.

## 2. Materials and Methods

### 2.1. Sampling Site, Collection and Storage

Biomass of *Gelidium corneum* was collected on the rocky coast of São Martinho do Porto (39°30′38.8″ N−9°08′36.2″ W), Leiria, Portugal once each season. The raw material was quickly taken to the laboratory, in dark cooled boxes, where it was washed in seawater to remove epiphytic organisms such as bacteria, vertebrates, and algae, among other debris. Thereafter, *G. corneum* was stored at −20 °C until further use. 

### 2.2. Biomass Drying Process

To determine the biochemical profile and prepare the extracts, *G. corneum* biomass was thawed at room temperature, in perforated laboratory trays, and placed in a ventilated oven (Model FD 115 Binder, Tuttlingen, Germany) at 25 °C until completely dry, usually for 24 h. The dry biomass was then milled using a blender (Krups, Solingen, Germany) and a coffee grinder (GVX212 Krups, Solingen, Germany), obtaining a fine powdery biomass.

### 2.3. Aqueous Extraction

The aqueous extraction (EA-PQ) used a dry alga:solvent ratio of 1:50 (g/mL) with the help of a Soxhlet system for 4 h using the percolation method adapted from Fatima and coworkers [48]. The algal biomass was evenly placed on laboratory paper to make the cartridge that was placed inside the Soxhlet apparatus. The respective volume of water was added to the extraction flask, always keeping in mind the 1:50 ratio, along with glass beads to avoid heavy foaming. The heating mantle was adjusted to the boiling point of water. The extract was freeze-dried and stored at room temperature until use.

### 2.4. Proximate Composition

#### 2.4.1. Determination of Moisture and Ash Content

Moisture and ash contents were determined according to the official AOAC standard methods [49]. Moisture content was determined using fresh algal biomass, which was weighed after blot drying and then incubated at 105 °C for 48 h. Moisture content was expressed as a percentage of fresh weight (fw). Ash content was determined by incinerating dry biomass in a muffle furnace (B170, Nabertherm, Lilienthal, Germany), with a 4 h heating ramp reaching a threshold of 525 °C for 5 h, after which it was allowed to cool to constant weight. The results were expressed as a percentage of dry weight (dw).

Iodine, lead, tin, arsenic, cadmium, and mercury contents were determined by inductively coupled plasma mass spectrometer (ICP-MS) and were outsourced. These analyses were performed in early June and late November to allow an approximate seasonal assessment. 

#### 2.4.2. Total Protein Content 

The total protein content was determined by the Kjeldahl method [49] and was estimated using a conversion factor of 5 specific to red seaweeds [50]. Seaweed samples (0.5 g) were digested with 25 mL of 97% sulfuric acid and two catalyst tablets (VWR CHEMICALS, Pennsylvania, USA) for 30 min at 200 °C followed by 90 min at 400 °C in a digestor system (Digestor2006, Foss, Hillerød, Denmark). The samples were cooled at room temperature and 80 mL of water was added. The samples were then distilled under alkaline conditions (Kjeltec2100, Foss, Hillerød, Denmark). The resulting distillate was collected in 4% boric acid solution and ammonia was quantified by titration with 0.1M hydrogen chloride using a mixture of methyl red and bromocresol green as an indicator. The protein content was calculated according to Equation (1).

(1) $$\mathrm{Total}\;\mathrm{protein}\;\left(\%\right)=\frac{\left(V_{s}-V_{b}\right)\times n\times 5\times 0.014}{m}\times 100$$

where V*_s_* represents the volume of HCL (mL) used in the titration of the sample, V*_b_* represents the volume of HCL (mL) used in the titration of the blank solution (prepared in absence of algae biomass), n corresponds to the concentration of the HCL solution used in the titration (M), and m corresponds to the initial mass (g) of the dried seaweed sample.

#### 2.4.3. Total Carbohydrates

The total carbohydrate content was determined using an adaptation of the method of Dubois et al. [51]. Biomass powder (5 mg) was added to 3 mL of H_2_SO_4_ 1M and the samples were placed in a water bath at 90 °C for 1 h. After cooling to room temperature, the samples were centrifuged at 805 g for 2 min. A volume of 0.5 mL of phenol 5% and 2.5 mL of 96% sulfuric acid were added to 1 mL of the sample. After cooling to room temperature, 5 mL of water were added. The absorbance was measured at 485 nm by an UV-Visible Spectrophotometer (Evolution201, Waltham, MA, USA). Total carbohydrate concentration was calculated by interpolation of a galactose calibration curve (Abs 485 nm versus galactose concentration (mg·mL^−1^)). The total carbohydrate content was expressed as percentage of dry weight, according to Equation (2).

(2) $$\mathrm{Total}\;\mathrm{Carbohydrates}\;\left(\%\right)=\left(\frac{\left(\frac{y-b}{a}\right)\times \;V}{m}\right)\times 100,$$

where *y* is the absorbance at 485 nm, *b* is the y-intercept value, *a* is the slope, *m* is the mass of the dried seaweed sample (mg), and V is the total volume of hydrolysate (mL).

#### 2.4.4. Total Lipid Content 

Lipid content was determined using the Folch method [52] with modifications. Approximately 1 g of dried biomass was mixed with 0.8 mL of water and 5 mL Folch reagent, consisting of a 2:1 ratio of CHCl_3_:MeOH. The mixture was vortexed for 5 min, and then 1.2 mL of 0.8% NaCl was added, and the mixture was vortexed for a further 2 min. The mixture was centrifuged at 4637× *g* for 10 min at 4 °C. The resulting lower phase was collected in a weighed evaporator flask through an anhydrous sodium sulfate column. After adding 5 mL of CHCl_3_ to the remaining mixture, it was centrifuged, and the chloroform phase was collected to the respective evaporator flask. The flask containing the lipidic residue was weighed and the total lipid content was expressed as a percentage and calculated according to Equation (3).

(3) $$\mathrm{Total}\;\mathrm{Lipid}\;\mathrm{Content}\;\left(\%\right)=\frac{Fw-Iw}{Sw}\times 100$$

where *Fw* is the mass of the flask and the lipid residue (g), *Iw* is the mass of the evaporator flask (g), and *Sw* is the mass of the dried seaweed sample (g).

#### 2.4.5. Fatty Acid Analysis

Fatty acid analysis was performed by an adaptation of the method described by Lepage and Roy [53]. To obtain the fatty acid methyl esters (FAMEs), 50 mg of fresh biomass was added to 2 mL of 2% sulfuric acid and the samples were vortexed and placed in a water bath at 80 °C for 2 h. Subsequently, 1 mL of ultrapure water and 2 mL of n-hexane were added, and the samples were vortex-stirred for 1 min. After centrifugation at 129× *g* for 1 min, the organic upper phase was collected in Gas Chromatography (GC) vials. The total fatty acid profiles were obtained by GC quantification of FAMEs in a Finnigan Ultra Trace GC equipped with a Thermo TR-FAME capillary column (60 mm × 0.25 mm ID, 0.25 µm film thickness), an autosampler AS 3000 from Thermo Electron Corporation, and a flame ionization detector (FID). The temperatures of the detector and injector, working in splitless mode, were 280 and 250 °C, respectively. The temperature gradient of the oven was 100 °C (1 min) with an increase of 10 °C min^−1^ until 150 °C, a second increase of 4 °C min^−1^ until 200 °C, and a third increase of 2 °C min^−1^ until a temperature of 235 °C was reached. Helium was used as a carrier gas at a flow rate of 1.2 mL min^−1^ and the FID detector was supplied with synthetic air and hydrogen at flow rates of 350 and 35 mL min^−1^, respectively. Each fatty acid was expressed as a percentage of the total peak area.

### 2.5. Indexes of Lipid Quality

From the data on the fatty-acid composition, we calculated the following indexes.

The atherogenicity index (AI index), which evaluates the risk of atherosclerosis, through the ratio between the pro-atherogenic fatty acids and the main classes of unsaturated fatty acids, is determined according to the following equation [54]:

(4) $$\mathrm{AI}\;=\frac{\Sigma (C12:0+\;C14:0+C16:0)\;}{\Sigma (\mathrm{n-6}+\mathrm{n-3})+\;\Sigma \mathrm{MUFA}}$$

The thrombogenicity index (TI index) relates pro-thrombogenic fatty acids (SFA) to anti-thrombogenic fatty acids (PUFA) and is the most widely used ratio to evaluate the influence of diet on cardiovascular health [54]. It is calculated according to the following equation:

(5) $$\mathrm{TI}\;=\frac{\Sigma (C14:0+\;C16:0+C18:0)}{0.5\times \;\mathrm{MUFA}\;+0.5\times \;\mathrm{n-6}+3\times \;\mathrm{n-3}+\frac{\mathrm{n-3}}{\mathrm{n-6}}}$$

The ratio n-6/n-3 balances the precursors of anti-inflammatory (n-3) and pro-inflammatory (n-6) metabolic eicosanoids [55].

Finally, the hypo- and hypercholesterolemic ratio (h/H ratio) was developed as an accurate measure of the effect of fatty acid composition on cholesterol [55] and was calculated according to the following equation:

(6) $$h/H\;=\frac{C14:0+\;C16:0\;}{\;\mathrm{MUFA}+\;\mathrm{PUFA}\;}$$

### 2.6. Agar Extraction and Quantification

Agar extraction and quantification were performed by an adaptation of the freeze–thaw method of Martínez-Sanz et al. [56]. The samples were hydrated in distilled water and immersed in double-distilled water, keeping in mind a 1:150 ratio of biomass and water, and kept in a water bath at 80 °C for 4 h. The samples were filtered into a 100 mL container, and the filtrate was frozen and thawed while removing excess water. This was followed by a total precipitation, through ethanol 96% for 24 h, and the colloid fraction was then placed in a drying chamber at 60 °C. The amount of agar was measured as percentage of dw.

### 2.7. Antioxidant Capacity

#### 2.7.1. DPPH Radical Scavenging Assay 

The 2,2-Diphenyl-1-picrylhydrazyl (DPPH) radical scavenging assay was performed by an adaptation of the method described by Brand-Williams et al. [57]. A volume of 1 mL of the aqueous extract (0.6 mg mL^−^^1^ in phosphate buffer pH 5.5) was added to 500 µL of phosphate buffer and 500 µL acetonitrile. Briefly, the DPPH radical solution was prepared with a concentration of 0.1 mg mL^−^^1^ in acetonitrile, 1 mL was added to each extract, and finally the mixture was vortexed. The test tubes were then incubated in the dark at room temperature for 30 min and the absorbance was read at 517 nm by an UV-Visible Spectrophotometer (Evolution201, Waltham, MA, USA). DPPH inhibition was calculated using the following formula:

(7) $$\mathrm{DPPH}\;\mathrm{inhibition}\;\left(\%\right)=\frac{A_{B}-A_{S}}{A_{B}}\times 100$$

where *A_B_* is the absorbance of the blank and *A_S_* is the absorbance of the sample, both at 517 nm. 

#### 2.7.2. Ferric Reducing Power Assay (FRAP)

The ferric reducing power assay was determined using the method reported by Adão et al. [58]. A volume of 1.5 mL of distilled water was added to 1 mL of the aqueous extract (0.6 mg mL^−1^) and 0.5 mL of a previously prepared [FeIII(Phen)3]Cl_3_ solution at 3.3 mM. The mixture was vortexed and incubated in the dark at room temperature for 30 min. Subsequently, absorbance was measured at 510 nm by an UV-Visible Spectrophotometer (Evolution201, Waltham, MA, USA). Ascorbic acid was used as a standard and through the calibration curve, the results were expressed as ascorbic acid equivalent (mg AAE^−1^). 

#### 2.7.3. ABTS Radical Scavenging Assay 

The 2,2′-Azino-bis(3-ethylbenzothiazoline-6-sulfonic acid) diammonium salt (ABTS) radical scavenging assay was performed by an adaptation of the method described by Nenadis et al. [59]. The ABTS radicals were obtained by the oxidation of 2,2′-azino-bis (3-ethylbenzothiazoline-6-sulfonic acid) (ABTS) (Alfa Aesar, Tewksbury, MA, USA) with potassium persulfate (Alfa Aesar, Tewksbury, MA, USA). A quantity of 47 mg of ABTS and 12.8 mg of potassium persulfate were added to 250 mL of distilled water and the reaction was placed in the dark at room temperature for 16 h. A volume of 2 mL of the ABTS radical solution was added to 2 mL of aqueous extract sample (0.6 mg ML^−1^ in distilled water) and 2 mL of distilled water. Each sample was prepared individually, stirred for 1 min and absorbance was measured at 734 nm by an UV-Visible Spectrophotometer (Evolution201, Waltham, MA, USA). The ABTS radical scavenging activity was calculated using the following formula:

(8) $$\mathrm{ABTS}\;\mathrm{radical}\;\mathrm{scavenging}\;\mathrm{activity}\;\left(\%\right)=\frac{A_{B}-A_{S}}{A_{B}}\times 100$$

where *A_B_* is the absorbance of the blank and *A_S_* is the absorbance of the sample, both at 734 nm. 

#### 2.7.4. Total Phenolic Compound Assay (TPC)

The TPC assay was performed using an adaptation of the Folin-Ciocalteau method by Singleton and Rossi [60]. The reaction was carried out in a 96 well plate, using 2 µL of the aqueous extract, at a concentration of 5 mg mL^−1^, with 158 µL of distilled water, 10 µL of Folin reagent, and 30 µL of a 20% sodium carbonate solution. The plate was then incubated in the dark and at room temperature for 1 h, and the absorbance was measured at 755 nm. Gallic acid was used as the standard and the results were expressed as gallic acid equivalent (mg GAE g^−1^). 

### 2.8. Statistical Analysis

All measurements were performed in triplicate, except for carbohydrate analysis which was *n* = 4. Results are expressed as mean ± standard deviation. All tests were performed considering the significance level at 5% (*p*-value < 0.05). Shapiro-Wilk test and Levene’s *F*-test were, respectively, used to test normality and variance of homogeneity. After ensuring that all requirements were validated (data normality and homogeneity of variance), analysis of variance (ANOVA) and *t*-tests were performed. If *F*-values showed significance, a comparison was made, using Tukey’s Honest Significant Difference HSD test. Whenever the data did not meet the assumptions, the nonparametric Kruskal-Wallis and Games-Howell tests were used. Statistical analysis was performed using IBM SPSS statistical software, version 21.0 (IBM Corporation, Armonk, NY, USA).

## 3. Results

### 3.1. Proximate Analysis

The analysis of the *Gelidium corneum* biomass was carried out each season at a single collection site. Measurements for moisture, ash, protein, lipid, carbohydrates, and agar are shown in Table 1.

We found average moisture values of 56.01 ± 2.96% dw. These values were higher in autumn (63.19 ± 0.43% dw) and lower in winter (45.61 ± 8.22% dw). The results of statistical analysis, established by the post hoc Games-Howell test, revealed significant differences between summer and autumn when compared to the spring biomass (*p*-value = 0.006 for both tests). Between the summer, autumn, and winter biomass, the differences were non-significant (*p*-value > 0.05). As for ash content, the results showed that *G. corneum* had an average ash content as high as 14.12 ± 0.85% dw in autumn. According to the Tukey’s multiple comparison test there was a significant difference between summer and winter (*p*-value = 0.015).

The total protein content showed significant variations over the seasons, peaking in autumn with a value of 16.25 ± 0.32%, and the lowest protein content in visible in spring (10.44 ± 0.19%). According to the Tukey’s multiple comparison test there was a significant difference between spring and the remaining seasons (*p*-value < 0.05 for all tests), summer and the remaining seasons (*p*-value = 0.006 and 0.023 for the autumn and winter, respectively), whereas the autumn and winter seasons showed non-significant differences in their protein values. 

The results showed low levels of total lipids, but within the expected range for red seaweeds, with a minimum of 0.93 ± 0.04% in winter and a maximum of 2.75 ± 0.28% in spring. According to the Tukey’s multiple comparison test there was a significant difference between the winter and the remaining seasons (*p*-value < 0.05 for all tests) and between the summer and spring biomass (*p*-value = 0.016).

Regarding agar, the maximum concentration obtained was in winter with a value of 8.70 ± 0.97%, but there were no statistically significant changes over the seasons as determined by one-way ANOVA (F (3,8) = 1.213, *p*-value = 0.366). 

Regarding the carbohydrate content, the values showed a maximum production in the spring with a value of 39.50 ± 3.29% and a minimum in autumn with a value of 24.84 ± 5.06%. There was a slight, yet non-significant decline in summer when compared to spring biomass as assessed by Tukey’s multiple comparison test (*p*-value = 0.058) and a significant decrease in autumn when compared to spring biomass (*p*-value = 0.007). Winter showed a significant difference with autumn biomass (*p*-value = 0.008).

The fatty acid (FA) profile of *G. corneum* collected in each season is shown in Table 2. Total saturated fatty acids (SFA) were the most abundant group, with values ranging from 46.36 ± 0.70% in summer to 75.12 ± 0.67% in winter. Total monounsaturated fatty acid (MUFA) ranged from 8.08 ± 0.34 in summer to 11.06 ± 1.17 in autumn. Being low in lipids, polyunsaturated fatty acids (PUFA) are abundant mainly during summer, reaching values of 42.56 ± 1.04%. These are significantly higher than the PUFA percentages in the other seasons, especially in winter when PUFA are as low as 13.83 ± 0.36%. Myristic acid, palmitic acid, palmitoleic acid, oleic acid, total SFAs, MUFAs, PUFAs, total n-3 and n-6 showed statistically significant seasonal variations (*p*-value < 0.05).

As expected, palmitic acid was the most abundant FA in all seasons, with values ranging from 42.79 ± 0.33% total in summer to 63.33 ± 0.99% in winter. According to the post hoc Games-Howell test, summer values of palmitic acid were statistically different when compared to spring biomass (*p*-value = 0.007), autumn values were statistically different when compared to spring and summer values (*p*-value = 0.004 for both tests), and winter values showed statistical differences compared to all seasons (*p*-value < 0.005). There are clear statistical differences among seasons, and the proportions of SFAs were set above common values for red seaweeds in autumn and winter (SFA > 50.35 ± 10.10%). Oleic acid is the most abundant MUFA present in the biomass throughout the seasons, with values varying 5.47 ± 0.06% in summer and 7.56 ± 0.28% in autumn, with statistical differences; according to Tukey’s multiple comparison test, the summer biomass showed a statistical difference when compared to spring (*p*-value = 0.001), whereas autumn and winter biomass showed statistical differences in comparison with summer (*p*-value < 0.005). Arachidonic acid (n-6) and eicosapentaenoic acid (n-3) are the two main PUFA present in *G. corneum*.

### 3.2. Biomass Quality

The AI index values ranged from 0.93 in summer to 2.89 in winter, with significant differences along the seasons. According to the post hoc Games-Howell test, the AI index showed a statistical difference in summer when compared to spring (*p*-value = 0.005), also in autumn when compared to spring and summer values (*p*-value = 0.018 and 0.013, respectively), and in winter when compared to the remaining seasons (*p*-value < 0.05). The TI index showed exactly the same pattern, with the lowest value in summer and the highest in winter. According to Tukey’s multiple comparison test, summer biomass showed statistically significant differences when compared to spring (*p*-value = 0.004), whereas autumn and winter TI values showed statistical differences among the remaining seasons (*p*-value < 0.05). These data indicate that the winter biomass showed higher atherogenic and thrombogenic indices, and summer the lowest, being thus the healthiest biomass. The n-6/n-3 ratio is very low, always close to 1, although with some significant statistical differences, being highest in autumn and lowest in the spring and summer. According to Tukey’s multiple comparison test, autumn showed a statistically significant difference when compared to all the other seasons (*p*-value < 0.05). Finally, the h/H ratio was in line with the other indexes, showing the healthiest value in summer and the lowest in winter, with 1.08 and 0.35, respectively. According to Tukey’s multiple comparison test, there were significant differences among all the seasons studied (*p*-value < 0.05, for all tests and all seasons).

The concentration of contaminants is shown in Table 3. These compounds are present in low concentrations, with arsenic being the most abundant, with 1.60 ± 0.28 mg kg^−^^1^ dw in autumn, but below the maximum levels established by the Commission Regulation (EU) 2015/1006, amending Regulation (EC) No 1881/2006, regarding maximum levels of inorganic arsenic in foodstuffs. The values for lead and cadmium are below these maximum levels and mercury and tin were not detectable. For iodine, values were always high. As determined by one-way ANOVA there were statistically significant differences between spring and autumn values for iodine content (F (1,4) = 13.298, *p*-value = 0.022) and no statistically significant differences for lead content (F (1,4) = 0.213, *p*-value = 0.668), arsenic (F (1,4) = 1.350, *p*-value = 0.310) and cadmium (F (1,3) = 0.360, *p*-value = 0.591) contents.

### 3.3. Antioxidant Capacity of Aqueous Extract of Gelidium Corneum

**Figure 2:**
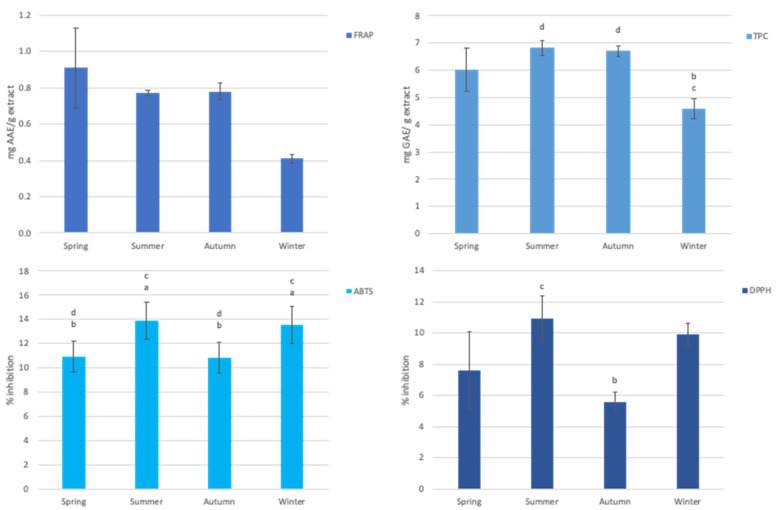
Seasonal antioxidant activity of the aqueous extracts of *G. corneum*, assessed through I–FRAP assay; II–TPC assay; III–ABTS assay; IV–DPPH assay. Lower case letters indicate significant differences when compared with the values of spring (**a**) summer (**b**) autumn (**c**) and winter (**d**) harvests. Top left: FRAP expressed as mg ascorbic acid equivalent (mg AAE g^−1^), top right: Total phenolic content, expressed as gallic acid equivalent (mg GAEg^−1^), bottom left: ABTS antioxidant assay expressed as percentage of inhibition, bottom right: DPPH antioxidant assay, expressed as percentage of inhibition.

The seasonal variation of the antioxidant activity of the aqueous extracts of *G. corneum* is shown in Figure 2. Although results were not expressive, it was possible to observe variation within the studied seasons. In the FRAP assay, there was a maximum of 0.91 ± 0.22 AAE.g^−^^1^ of extract corresponding to spring biomass, but no statistical seasonal variation was observed. The TPC assay exhibited similar values for the spring, summer, and autumn biomass (up to 6.82 ± 0.26 GAE.g^−^^1^). However, the winter biomass showed a statistical difference, according to Tukey’s multiple comparison test, when compared to the summer and autumn biomass (*p*-value = 0.006 and 0.008, respectively), producing a minimum value of 4.59 ± 0.37 GAE.g^−^^1^ extract. The DPPH assay and ABTS assay showed similar variations in the seasons under analysis, with inhibition values ranging from 5.57 ± 0.62% to 10.89 ± 1.46% and 10.85 ± 1.25% to 13.90 ± 1.54%, respectively. In the ABTS assay the inhibition values were significantly higher in summer and winter, compared to the other seasons. According to Tukey’s multiple comparison test, the summer biomass showed a statistically significant change when compared to the spring (*p*-value = 0.006), the values for autumn showed statistical differences when compared to the summer biomass (*p*-value = 0.005), and the winter biomass showed a statistically significant change when compared to the spring and autumn values (*p*-value = 0.013 and 0.011, respectively). In the DPPH assay there were statistically significant differences, shown by Tukey’s multiple comparison test, shown only in the autumn biomass regarding the summer biomass (*p*-value = 0.032).

## 4. Discussion

It is well known that seaweeds can undergo fluctuations in their metabolism that lead to changes in their nutritional composition, driven by abiotic and biotic factors, such as temperature, pH, and light, among others [18,42,61]. To determine the best time to harvest algal biomass, depending on its intended application, it is essential to gather information about variations in the production of bioactive compounds. So far, it is known that solar radiation, water temperature, and depth, among other factors, influence the chemical composition of *G. corneum* [62,63].

The moisture content varied slightly, but with significant differences between the seasons under study. Moisture is an important factor when considering the quality and shelf-life of new seaweed products, as a high moisture content may fuel the growth of microorganisms [64]. These values reached a maximum of 63.19 ± 0.43%, much higher when compared to *Gelidiella acerosa* (8.71%) [65] and *Palmaria palmata* (4%) [66] but still lower than the value found for *Mastocarpus stellatus* (74.1%) [67]. When considering the shelf-life, dehydration of biomass is the most common preservation process, considering the high moisture content of seaweed. 

In *G. corneum*, although ash values were low compared to other *Gelidium* species such as *G. pusillum* (21.2% dw), it is still within the expected range for red seaweeds (5.8–46.2%) [68]. In our study, this value reached a maximum of 14.12 ± 0.85%, indicating a relatively low mineral richness when compared to *Gracilaria vermiculophylla* (formerly *Agarophyton vermiculophyllum*) and *Gelidium pusillum* [68,69]. 

Macroalgae are widely studied due to their nutritional composition, and regarding lipid content, there is a particular focus on brown and red algae [24,70]. Methods based on chloroform/methanol solvents are used as standard methods, the Folch method being one of them [71]. The results as to total lipid content agreed with the expected values considering seaweeds (1–6%) and specifically red macroalgae (1–4%) [72,73]. Polar lipids are the most abundant and have a wide range of cellular functions, such as membrane structure [74]. They are mostly glycerophospholipids and, in some species, can represent up to 90% of the total polar lipids [73]. Although, as noted above, algae have a low total lipid content, *G. corneum* produced higher values than *Palmaria* sp. (1.8 ± 0.14%), *Undaria pinnatifida* (1.05 ± 0.01%), or *Himanthalia elongata* (0.97 ± 0.07), which are edible seaweeds [75]. There are several studies that recognize maximum values of lipid production scattered through the year among different algae. In Kendel et al. [72] and Denis et al. [76], *Grateloupia turuturu* produced maximum lipid values in the summer. In Nelson et al. [73] and Kumar et al. [77], they recognized that *Chondracanthus canaliculatus* and *Kappaphycus alvarezii* produced more lipids in the winter. In our study *G. corneum* showed statistical differences of total lipid content in winter and these low values may be due to the use of lipid reserves for growth, as it has already noted by Ngan and Price [78]. 

The AI and TI indexes have been widely used to evaluate seaweeds, related to coronary heart disease. Regarding the AI index, seaweeds have a wide range of values from 0.03 to 3.58 [79] and red seaweeds show the highest values, from 0.38 to 2.87 [80]. These values are consistent with our data. For *Gelidium* sp. a value of 1.61 was reported [80], which is similar to the value we found for autumn (1.94), but very different values were recorded for summer (0.93) and winter (2.89). The same pattern was found for the TI index. Kumar et al. [77] reported a value of 1.83 for *G. corneum*, similar to the one we found for autumn (1.57), but a much lower value was obtained for the summer biomass (0.58) and a much higher value was obtained for the winter biomass (2.36). As to the n-6/n-3 index, evaluating the level of pro-inflammatory/anti-inflammatory precursors, we again found values similar to those reported by Kumar et al. (1.02) [77]. The World Health Organization recommends a diet with a n-6/n-3 lower than 10 to prevent inflammatory, cardiovascular, and nervous system disorders. *G. corneum* certainly has a much lower value with some seasonal variations (0.88 to 1.23), so this index is always close to 1.0 [81]. Finally, the h/H index measures the effect of fatty acid composition on cholesterol. For this index, a higher value is better, so the best season is summer with 1.08. Seasonality is once again an important factor, with very different values, ranging from 0.35 in winter to 1.08 in summer. These values are similar or lower than those presented for *Porphyra* sp. (1.1–1.3), *G. vermiculophylla* (0.63–2.02), *Gracilaria gracilis* (2.1–2.6), and *Chondrus crispus* (8.4–9.2) [69]. Thus, environmental conditions have an impact on the production of different FA, with PUFA being produced mainly during summer and SFA during winter. This seasonal variation has been registered before for red seaweeds [55,69,82], but with an increase in PUFA during summer. The mild temperatures recorded in Portugal, in the central area, may have less impact on PUFA production, and other environmental conditions such as nutrient availability, light intensity during summer, salinity, and the physiological state of the thalli, or the life cycle of the species itself, may influence the production of these primary metabolites [46,83]. Thus, regarding the fatty acids pattern, considerable changes are found for the biomass in different seasons of the year, with the summer biomass being the healthiest one. 

To avoid the consumption of chemical additives, seaweeds also present themselves as a healthy and natural alternative. There have been reports of in vitro digestion values of red algae proteins being higher than those of green and brown algae proteins. The high in vitro digestion values (83–87%) are comparable with the values for vegetables (72–92%) and fruit (72–92%) [84]. The protein content of *G. corneum* is very similar to that found for *G. microdon* (15.18%) [85], which is commonly used in the agar industry [86] and higher than that to the brown algae *Gongolaria abies-marina* (formerly *Cystoseria abies-marina*) (% <6.8%) and *Fucus spiralis* (% <11%), which are used for phlorotannin production [87,88]. It is, however, slightly lower compared to the red algae *G. vermiculophylla* (% ≈22), *Osmundea pinnatifida* (% ≈20)*, Porphyra* sp. (% ≈25), and *Pterocladiella capillacea* (% ≈20), which are traditionally used in the food industry [69,85,89,90]. These data corroborate that those seaweeds are an interesting source of protein. Nonetheless, there are statistically significant variations of protein production with a peak in autumn/winter and a trough in spring/summer, similar to the values reported for the red algae *Palmaria palmata* [91]. Martinez and Rico [91] state that protein accumulation is associated with the development of reproductive structures. The lower values in spring and summer may also be due to the loss of phycobiliproteins due to extensive UV light exposure and low availability of nutrients [92].

*G. corneum* is used mostly in the agar industry for its high-quality agar, the agar extraction yielded values ranging from 5.99–8.70% dw, which is relatively low when compared to *G. elegans*, *G. serrulatum,* and *G. floridanum* (21%, 33%, and 31.7% respectively) [93,94], but within the values reported by Li et al. (3.3–18.15%) [95] and close to those reported by Martínez-Sans et al. (10–12%) [56] for *G. corneum* (as *G. sesquipedale*).The comparison of these extractions is very complex due to the variety of extraction parameters, such as the water bath time and biomass to water ratio [96,97]. The main discussion seems to be the extraction method employed, with highly variable values being reported for different methods, hence the method employed by us most certainly requires refinement [56,98].

Among the many components of macroalgae, carbohydrates are the most abundant and are the main source of energy in most human diets. Moreover, due to their physical and chemical properties, they can be used for production of biofuels and are sought after by the cosmetic industry, among others [99,100]. *G. corneum* gives off high carbohydrate values (up to 39.5%) when compared to other red macroalgae, such as *p. palmata* (25%) [101] or *Crassiphycus corneus* (formerly *Gracilaria cornea*) (36%) [102]. The maximum production values for brown macroalgae, which are a focal group to produce biofuel, have a maximum production of carbohydrates usually in autumn to allow an accumulation of nutrients that will be used in winter for protein synthesis, reproduction, and growth [103]. These peaks in carbohydrate production vary within the many species of macroalgae due to their different life cycles and abiotic fluctuations. According to Santelices [104], several species of *Gelidium* tend to store carbohydrates in winter in order to spend the stored energy as a means of reproduction and growth, as is also noticeable in the current study as the highest values obtained for carbohydrate content are in the winter and spring seasons.

Heavy metals are present in the environment through anthropogenic and natural sources. Their concentration in the environment varies depending on the characteristics of the region in question and the quality of the water in which the seaweeds grow. They can be taken up by seaweeds and, as there is no known homeostasis mechanism for them in the human body, ingestion of macroalgae may result in an intake of these hazardous compounds that may lead to neurotoxic and/or oncological diseases [105]. As noted above, the concentrations found for lead, cadmium, and mercury are below the recommended level set by Commission Regulation (EU) 1881/2006, respectively 3.0, 3.0, and 0.1 mg kg^−^^1^. As to tin, there is no specific limit for seaweeds, but the maximum level set for other foods is 50 to 200 mg kg^−^^1^, much higher than the values found in our samples. Arsenic is regulated by directive 2002/32/EC, which established a maximum limit for inorganic arsenic of 2 mg kg^−^^1^ [106], also higher than the concentration found in our samples (1.5 and 1.6 mg kg^−^^1^). Although below the European legal limit, this is the most predominant heavy metal in our samples. However, this is a poor indicator of health risks related to food products with macroalgae. Most of the arsenic present in the macroalgae is assimilated to organic molecules such as arsenosugars and is, therefore, less harmful than inorganic forms of arsenic, which are toxic [30].

The iodine content in *G. corneum* varied significantly within the two months analyzed. Seaweeds are known to be a food source of iodine, which is required throughout life and is related to the proper development of cognition function in children. *G. corneum* has a higher iodine content when compared to Nori (*Porphyra/Pyropia*), a typically edible red macroalgae, but in autumn the iodine content is low when compared to other edible seaweeds such as Kombu (*Laminaria*) and Wakame (*Undaria*). [107]. The upper limit established by the European Commission Recommendation 2018/464 of iodine uptake is 600 µg day^−^^1^ for adults and 200 µg day^−^^1^ for children aged 1–3 years. Therefore, according to the results obtained in this study, to reach this threshold, an adult would have to ingest approximately 2.54 g and 4 g, for the spring and autumn biomass, respectively, to reach the daily iodine requirements. 

*G. corneum* is stated as a species with low antioxidant capacity, and the values in our study fall within the range found by Matos et al. [108]. In fact, for DPPH our data are rather low (up to 10.89%) when compared to the values obtained for other red algae such as *G. amansii, Neopyropia tenera* (formerly *Porphyra tenera*), *Scinaia okamurae*, or *Lithophyllum okamurae,* among others, whose inhibition rates in the DPPH assay fall between 20 and 30% inhibition [109]. These are three times the highest value obtained in our study from the summer biomass. These values are, however, high when compared to the aqueous extracts of the marine alga *Caulerpa chemnitzia* (formerly *Caulerpa peltata*)*, Gelidiella acerosa, Padina gymnospora* and *Sargassum wightii,* which show inhibition values of less than 8% [110]. As for the total phenolic content, it is similar to other red algae reported by Heo and coworkers [111], being less than 10 GAE.g^−^^1^. The results obtained for the FRAP assay were lower to those of several brown algae such as *Turbinaria ornata, Hydroclathrus clathratus,* and *Sargassum aquifolium* (formerly *S. echinocarpum*); green algae such as *Gayralia oxysperma* and *Chaetomorpha antennina;* and other red algae such as *Wilsonosiphonia howei* (formerly *Polysiphonia howei*)*, Gymnogongrus durvillei* (formerly *Ahnfeltiopsis concinna*), and *Pterocladiella capilacea* [111]. In another study by Matos and coworkers [108] they assessed the antioxidant activity of both aqueous and ethanolic extracts of *Gelidium corneum* (as *G. sesquipedale*). Both extracts produced low to no activity, and only the ethanol extract produced a 6.8% inhibition in the DPPH assay. The FRAP assay did not detect any antioxidant activity. Thus, our results are comparable with Matos et al. [108] aqueous extracts for the spring and autumn biomass and are higher for the values obtained for the summer and winter biomass. 

Although the aqueous extracts of *G. corneum* may not possess notable antioxidant activity, significant differences can be observed between seasons. This variation may be explained by fluctuations in the production of mycosporine-like amino acids (MAAs) that are produced by cyanobacteria and algae to protect DNA from UV radiation, which also have antioxidant activity. Their concentration may vary according to environmental factors [112,113]. 

## 5. Conclusions

The results obtained in the present study showed that the nutritional profile and antioxidant capacity of *G. corneum* have seasonal variation patterns. The results obtained indicate that the best time to harvest *G. corneum* depends on the component sought. For protein and carbohydrates, the best harvest seasons are autumn and spring, respectively. Regarding lipids and their associated fatty acids, although there are slightly more lipids in spring, the nutritional indexes indicate that summer is the best time to harvest this biomass. Regarding antioxidant activity, summer is also the best time to harvest *G. corneum*. 

Thus, with the available data *G. corneum* can be considered as having high nutritional value, being, therefore, a potential food source, which could be used in the food industry beyond agar extraction. The low content of heavy metals also encourages the use of this wild biomass as a food source. However, it would be interesting to check the digestibility, as a low digestibility value can compromise the potential benefits. Additionally, further chemical characterization, including secondary metabolites and minerals, will help to understand whether this interesting seaweed can be used in other seaweed-based products.

Finally, the cultivation of this species is of utmost importance to ensure year-round biomass availability and avoid the depletion of natural resources. 

## Figures and Tables

**Table 1:** Proximate composition of *Gelidium corneum*. Data are displayed as mean values ± standard deviation (*n* = 3), except for carbohydrates where *n* = 4). Lower case letters indicate significant differences within the values of spring ^a^, summer ^b^, autumn ^c^, and winter ^d^ harvests.

	Spring	Summer	Autumn	Winter
Moisture (% fw)	52.53 ± 1.99 ^bc^	62.69 ± 1.21 ^a^	63.19 ± 0.43 ^a^	45.61 ± 8.22
Ash (% fw)	12.68 ± 0.73	11.30 ± 0.84 ^d^	14.12 ± 0.85	13.21 ± 0.45 ^b^
Protein (%)	10.44 ± 0.19 ^bcd^	14.61 ± 0.71 ^acd^	16.25 ± 0.33 ^ab^	15.47 ± 0.21 ^ab^
Lipids (%)	2.75 ± 0.28 ^abd^	2.16 ± 0.10 ^ad^	2.57 ± 0.04 ^d^	0.93 ± 0.04 ^abc^
Agar (%)	5.99 ± 0.88	6.33 ± 2.43	6.01 ± 1.90	8.70 ± 0.97
Carbohydrates (%)	39.50 ± 3.29 ^c^	29.78 ± 1.60 ^d^	24.84 ± 5.06 ^ad^	39.08 ± 2.71 ^bc^

**Table 2:** Fatty acid composition in *Gelidium corneum* (as a percentage of total fatty acids, TFA). Data are presented as mean values ± standard deviation (*n* = 3). Lower case letters indicate significant differences when compared with the values of spring ^a^, summer ^b^, autumn ^c^, and winter ^d^ harvests.

		Spring	Summer	Autumn	Winter
Tridecyclic acid	C13:0	0.55 ± 0.18	0.60 ± 0.16	0.87 ± 0.28	0.67 ± 0.09
Myristic acid	C14:0	5.86 ± 0.24 ^bd^	4.13 ± 0.13 ^acd^	6.33 ± 0.14 ^b^	8.49 ± 0.17 ^abc^
Palmitic acid	C16:0	45.97 ± 0.53 ^bcd^	42.79 ± 0.33 ^acd^	56.07 ± 0.93 ^abd^	63.33 ± 0.99 ^abc^
Stearic acid	C18.0	2.26 ± 1.26	1.47 ± 1.00	1.46 ± 0.03	2.03 ± 0.18
Sum SFA%		55.05 ± 1.14 ^bcd^	49.36 ± 0.70 ^acd^	64.46 ± 0.90 ^abd^	75.12 ± 0.67 ^abc^
Myristoleic acid	C14:1	0.37 ± 0.13	0.44 ± 0.07	0.85 ± 0.00	0.52 ± 0.03
Palmitoleic acid	C16:1 n-7	1.46 ± 0.01 ^b^	0.899 ± 0.08 ^acd^	1.08 ± 0.23 ^b^	1.42 ± 0.12 ^b^
Oleic acid	C18:1 n-9	7.23 ± 0.55 ^b^	5.47 ± 0.06 ^acd^	7.56 ± 0.28 ^b^	7.48 ± 0.14 ^b^
Sum MUFA%		11.03 ± 0.27 ^b^	8.08 ± 0.34 ^acd^	11.06 ± 1.17 ^b^	11.05 ± 0.67 ^b^
Linoleic acid	C18:2 n-6	0.94 ± 0.38	0.75 ± 0.23	0.71 ± 0.07	0.52 ± 0.04
Arachidonic acid	C20:4 n-6	14.68 ± 1.48 ^bcd^	18.87 ± 0.81 ^acd^	10.98 ± 0.78 ^abd^	5.95 ± 0.18 ^abc^
Eicosapentaenoic acid	C20:5 n-3	17.64 ± 0.14 ^bcd^	22.68 ± 0.51 ^acd^	9.49 ± 0.28 ^abd^	6.87 ± 0.29 ^abc^
Sum PUFA%		33.92 ± 1.18 ^bcd^	42.56 ± 1.04 ^acd^	21.18 ± 1.07 ^abd^	13.83 ± 0.36 ^abc^
Nutritional Indexes					
Σ n-3		17.91 ± 0.13 ^bcd^	22.68 ± 0.51 ^acd^	9.49 ± 0.28 ^abd^	7.08 ± 0.23 ^abc^
Σ n-6		16.01 ± 1.07 ^bcd^	19.88 ± 0.61 ^acd^	11.9 ± 0.79 ^abd^	6.74 ± 0.16 ^abc^
AI index		1.15 ± 0.02 ^bcd^	0.93 ± 0.01 ^acd^	1.94 ± 0.10 ^abd^	2.89 ± 0.09 ^abc^
TI Index		0.82 ± 0.02	0.60 ± 0.01	1.64 ± 0.10	2.54 ± 0.05
n-6/n-3 ratio		0.89 ± 0.05 ^c^	0.88 ± 0.02 ^c^	1.23 ± 0.05 ^abd^	0.95 ± 0.02 ^c^
h/H ratio		0.87 ± 0.01 ^bcd^	1.08 ± 0.01 ^acd^	0.52± 0.02 ^abd^	0.35± 0.01 ^abc^

SFA—saturated fatty acids; MUFA—monounsaturated fatty acids; PUFA—polyunsaturated fatty acids.

**Table 3:** Heavy metals and iodine content of *G. corneum*. Data are displayed as mean values ± standard deviation (*n* = 2). Upper case letters indicate significant differences with the values of spring ^a^ and autumn ^c^ harvests.

Iodine and Contaminants (mg kg^−1^ dw)	Spring (June)	Autumn (November)
Lead	0.24 ± 0.12	0.52 ± 0.16
Arsenic	1.50 ± 0.1	1.60 ± 0.28
Cadmium	0.18 ± 0.02	0.12 ± 0.04
Mercury	<0.007	<0.007
Tin	<0.5	<0.5
Iodine	236.67 ± 0.12 ^c^	150.00 ± 0.16 ^a^

## Data Availability

The data presented in this study are available on request from the corresponding author (C.A.), upon reasonable request.
